# CACNA1C Gene rs11832738 Polymorphism Influences Depression Severity by Modulating Spontaneous Activity in the Right Middle Frontal Gyrus in Patients With Major Depressive Disorder

**DOI:** 10.3389/fpsyt.2020.00073

**Published:** 2020-02-25

**Authors:** Xiaoyun Liu, Zhenghua Hou, Yingying Yin, Chunming Xie, Haisan Zhang, Hongxing Zhang, Zhijun Zhang, Yonggui Yuan

**Affiliations:** ^1^ Department of Psychosomatics and Psychiatry, Zhongda Hospital, School of Medicine, Southeast University, Nanjing, China; ^2^ Department of Neurology, ZhongDa Hospital, School of Medicine, Southeast University, Nanjing, China; ^3^ Department of Clinical Magnetic Resonance Imaging, the Second Affiliated Hospital of Xinxiang Medical University, Xinxiang, China; ^4^ Department of Psychiatry, the Second Affiliated Hospital of Xinxiang Medical University, Xinxiang, China

**Keywords:** major depressive disorder (MDD), amplitude of low-frequency fluctuation (ALFF), right medial frontal gyrus (MFG), mediation effect, depression severity, CACNA1C

## Abstract

**Objectives:**

This study aimed to examine whether the CACNA1C gene rs11832738 polymorphism and major depressive disorder (MDD) have an interactive effect on the untreated regional amplitude of low-frequency fluctuation (ALFF) and to determine whether regional ALFF mediates the association between CACNA1C rs11832738 and MDD.

**Methods:**

A total of 116 patients with MDD and 66 normal controls (NCs) were recruited. The MDD and NC groups were further divided into two groups according to genotype: carriers of the G allele (G-carrier group, GG/GA genotypes; MDD, n = 61; NC, n = 26) and AA homozygous group (MDD, n = 55; NC, n = 40). MDD was diagnosed based on the *Diagnostic and Statistical Manual of Mental Disorders, Fourth Edition*. Depression severity was assessed using the Hamilton Depression Scale-24 (HAMD-24) at baseline and follow-up (after 2 and 8 weeks of treatment). All subjects underwent functional MRI (fMRI) scans at baseline, and the ALFF was calculated to reflect spontaneous brain activity. The interactions between MDD and CACNA1C single nucleotide polymorphism rs11832738 were determined using two-way factorial analysis of covariance, with age, sex, education, and head motion as covariates. We performed mediation analysis to further determine whether regional ALFF strength could mediate the associations between rs11832738 and depression severity, MDD treatment efficacy.

**Results:**

MDD had a main effect on regional ALFF distribution in three brain areas: the right medial frontal gyrus (MFG_R), the left anterior cingulate cortex (ACC_L), and the right cerebellum posterior lobe (CPL_R); CACNA1C showed a significant interactive effect with MDD on the ALFF of MFG_R. For CACNA1C G allele carriers, the ALFF of MFG_R had a significant positive correlation with the baseline HAMD-24 score. Exploratory mediation analysis revealed that the intrinsic ALFF in MFG_R significantly mediated the association between the CACNA1C rs11832738 polymorphism and baseline HAMD-24 score.

**Conclusions:**

A genetic variant in CACNA1C rs11832738 may influence depression severity in MDD patients by moderating spontaneous MFG_R activity.

## Introduction

Major depressive disorder (MDD) is a highly prevalent psychological condition characterized by a persistent low mood and anhedonia (1). The lifetime prevalence of MDD is approximately 17% (2), affecting approximately 350 million people worldwide (3, 4). According to recent data from 2019, the lifetime prevalence of MDD in China is as high as 3.4%, and it is estimated that approximately 44 million people suffer from this disease (5). More importantly, MDD is associated with substantial disability, mortality, and a high suicide rate, which places a heavy burden on patients, families, and the society, potentially making it the world's most economically burdensome illness by 2030 (6). Unfortunately, the current diagnostic criteria for MDD are mainly based on clinical symptoms, and the diagnostic consistency is very low (only 28%) (1). Poor consistency in diagnostic criteria can seriously affect the clinical efficacy of depression treatment: approximately 20% of patients with MDD fail to respond to standard antidepressant treatment, and up to 60% of patients with MDD still have residual symptoms after treatment (7). Therefore, it is very important to determine the pathophysiological mechanisms and to explore the objective biomarkers of depression in order to improve the consistency of diagnosis and treatment.

Imaging genetics is an emerging field aimed at identifying the associations between genetic variants and neuroimaging, bringing considerable promise in understanding the role of genes in determining the pathophysiological mechanism of MDD (8). Resting-state functional MRI (rs-fMRI) is one of the most commonly used neuroimaging techniques in imaging genetics studies. In rs-fMRI, spontaneous blood oxygen level-dependent (BOLD) low frequency fluctuations (0.01–0.08 Hz) were first suggested to have physiological significance by Biswal and colleagues, and this marker has been demonstrated to be closely related to spontaneous neural activities (9). The amplitude of low-frequency fluctuation (ALFF) based on voxel-wise analysis of the whole brain has been proposed as a quantitative measure of local BOLD signal variation due to regional spontaneous brain activity, which is an index reflecting the intensity of spontaneous neural activity within a specific frequency range, with no filtering at baseline (10).

Abnormal activity in frontotemporal regions (the cognitive and emotional control system in the cortico-limbic network) plays an important role in the pathophysiology of depression. The calcium channel, voltage-dependent, L type, alpha 1C subunit (*CACNA1C*) gene is considered to be involved in the development and plasticity of frontotemporal regions (11). The CACNA1C gene, located on chromosome 12p13.3, encodes the major L-type voltage-dependent calcium channel, Cav1.2 (alpha-1C subunit) (12). This channel is found in many types of cells, regulating the activity-dependent influx of calcium and is important for the normal functioning of the heart and brain cells (12). Evidence from animal studies, clinical studies, and genome-wide association studies supports the findings that CACNA1C is closely associated with depression (3, 13).

Many studies have focused on the CACNA1C single nucleotide polymorphism (SNP) rs1006737, which is located in the third intron of CACNA1C. This SNP has been demonstrated to have a close association with the minor allele A (adenine) and MDD (12, 14). To date, there have been few studies on other CACNA1C SNPs. However, rs11832738 is an SNP that is close to rs1006737, and they are both introns. Our research group used a multiple regression method and found that rs11832738 could significantly affect the anhedonia-associated grey matter network of MDD (15). Therefore, considering the results of our previous study, we hypothesized that this SNP may also be closely related to MDD. To the best of our knowledge, aside from our previous study, the associations between rs11832738 and MDD as well as other psychiatric disorders have not been studied. In this study, we explored the relationship between the polymorphism of the CACNA1C gene locus rs11832738 and MDD and investigated whether the gene locus could affect the severity and therapeutic effect of MDD by influencing spontaneous brain activity (reflected by ALFF values). We suspect that this gene locus affects MDD by influencing frontotemporal activities. Considering the complex etiology of MDD, we believe that it is critical to explore alterations in spontaneous brain activity and their associations with gene polymorphisms and depression severity, as well as possible therapeutic effects. Such exploration would help to elucidate the pathogenesis of MDD and clarify appropriate antidepressants to improve therapeutic effects and reduce the burden of depression.

## Methods

### Participants

A total of 182 participants were recruited, including 116 MDD patients (MDD group) and 66 normal controls (NC group). The MDD patients were recruited through the inpatient and outpatient departments of psychiatry in the Second Affiliated Hospital of Xinxiang Medical University (n = 61) and ZhongDa Hospital Affiliated with Southeast University (n = 55), while the NC subjects were recruited through media advertising and community posting and through completed MRI scans at the Second Affiliated Hospital of Xinxiang Medical University (n = 39) and ZhongDa Hospital Affiliated with Southeast University (n = 27). All participants were of Chinese Han ethnicity and right-handed. All participants signed informed consent forms, as approved by the local institutional review boards, and all study procedures adhered to the Declaration of Helsinki. The participants underwent MRI scans at baseline. All patients were in the acute phase of the disease and received antidepressant treatment depending on the clinical judgment of the empirical senior psychiatrists after the first scan and the first clinical scale assessment. Among the 116 patients with MDD, 51 experienced their first episodes without a history of antidepressant use, and the rest were recurrent patients with a history of antidepressant use. On searching the SNP database, we found that the frequency of the rs11832738 A allele was 72.5% in the East Asian population, while that of the G allele (guanine) was 27.5% (https://www.ncbi.nlm.nih.gov/snp/rs11832738#frequency_tab). According to our data, the frequency of the G allele was 22% in NC and 32.3% in MDD. Therefore, the G allele was suggested to be the rare risk allele. Accordingly, we further divided the MDD and NC groups into two subgroups: an AA homozygous group (MDD group, n = 55; NC group, n = 40) and a G-carrier group (GG/GA genotypes; MDD group, n = 61; NC group, n = 26).

### Inclusion and Exclusion Criteria

The inclusion criteria for the MDD group were as follows: individuals who (1) were aged ≥ 18 years; (2) met the criteria listed in the *Diagnostic and Statistical Manual of Mental Disorders, Fourth Edition*; (3) had a Hamilton Depression Scale-24 (HAMD-24) score ≥ 20; and (4) had no contraindication to MRI scanning. The exclusion criteria were as follows: individuals with (1) other major psychiatric disorders or neurodegenerative illness history; (2) substance abuse (drug, caffeine, nicotine, alcohol, or others), head trauma, or loss of consciousness; or (3) a cardiac or pulmonary disease which could influence the MRI scan.

NC subjects were required to have a HAMD-24 score of ≤7. The exclusion criteria included a history of neuropsychiatric disease, substance abuse or insobriety, or contraindications to MRI scanning.

### Clinical Evaluation

We used the HAMD-24 to assess depression severity at baseline and used the HAMD-24 reductive rate, calculated as (baseline HAMD score − follow-up HAMD score)/baseline HAMD score × 100%, to evaluate the effect of MDD treatment at the end of the 2- and 8-week treatments.

### Genotyping

DNA was extracted from blood using standard protocols. DNA genotyping was performed by Tianhao Biotechnology Company (Shanghai, China). SNP genotypes in CACNA1C genes (rs11832738) were determined using predesigned Illumina next sequencing and array technologies (Illumina Inc., San Diego, CA, USA). Hardy-Weinberg equilibrium (HWE) tests, linkage disequilibrium statistics, and allele and genotype frequencies were calculated using PLINK 1.9 software (16). The SNP did not deviate from the HWE (*r* = 0.78, *P* = 0.44).

### MRI Data Acquisition

MRI data were acquired using a Siemens 3.0 Tesla scanner with a 12-channel head coil (Siemens, Erlangen, Germany) in the Department of Clinical Magnetic Resonance Imaging at the Second Affiliated Hospital of Xinxiang Medical University and the affiliated ZhongDa Hospital of Southeast University. The heads of the participants were stabilized with a cushion to minimize head motion. Earplugs were used to reduce the scanner noise. High-resolution three-dimensional T1-weighted scans were recorded as magnetization-prepared rapid-acquisition gradient echo sequence (repetition time [TR] = 1,900 ms; echo time [TE] = 2.48 ms; flip angle [FA] = 9°; acquisition matrix = 256 × 256; field of view [FOV] = 250 × 250 mm^2^; thickness = 1.0 mm; gap = 0; time = 4 min 18 s, 176 volumes). The parameters of the rs-fMRI were as follows: TR = 2,000 ms, TE = 25 ms, FA = 90°, acquisition matrix = 64 × 64, FOV = 240 × 240 mm^2^, thickness = 3.0 mm, gap = 0 mm, 36 axial slices, 240 volumes, 3.75 × 3.75 mm^2^ in-plane resolution parallel to the anterior-posterior commissure line, and an acquisition time of 8 min. During scanning, the participants were instructed to lie still in the scanner, keep their eyes open, and refrain from falling asleep.

### MRI Image Preprocessing

For quality control, all image data were checked by two experienced radiologists. The rs-fMRI images were preprocessed using the Data Processing Assistant for Resting-State Function (DPARSF 2.3 advanced edition) MRI toolkit (17), which synthesizes procedures based on the resting-state functional MRI toolkit (REST, http://www.restfmri.net) and statistical parametric mapping software package (SPM, http://www.l.ion.ucl.ac.uk/spm). The first 10 time points were excluded to ensure stable longitudinal magnetization and adaptation to inherent scanner noise. The remaining 230 images were processed sequentially according to the following steps: (1) slice time was used with the 36th slice as the reference slice, corrected for temporal differences and head motion (participants with a head motion with maximum displacement greater than 1.5 mm in any direction [x, y, or z] or 1.5° of angular motion were excluded from the analyses); (2) T1 was co-registered to functional images and subsequently reoriented; (3) for spatial normalization, T1-weighted anatomic images were segmented into white matter, grey matter, and cerebrospinal fluid, and subsequently normalized to the Montreal Neurological Institute space using transformation parameters estimated with a unified segmentation algorithm (18). These transformation parameters were applied to the functional images, and the images were resampled with isotropic voxels of 3 mm; (4) spatial smoothing was conducted with a 4-mm full-width at half-maximum (FWHM) isotropic Gaussian kernel; (5) the linear trend within each voxel's time series was removed; (6) nuisance signals (white matter, cerebrospinal fluid signals, and head motion parameters calculated using rigid body six correction) and spiked regressors were regressed out; and (7) finally, temporal bandpassing (0.01–0.08 Hz) was performed to minimize low-frequency drift and filter high-frequency noise.

### ALFF Analysis

After preprocessing, ALFF was calculated using the DPARSF software. For a given voxel, the time series was transformed into the frequency domain, and the power spectrum was obtained. The square root of the power spectrum was subsequently computed and averaged across a predefined frequency interval. The average square root was taken as the ALFF, which was assumed to reflect the absolute intensity of spontaneous brain activity (17).

## Statistical Analyses

### Demographic and Clinical Data

Independent one-sample or two-sample t-tests or the chi-square tests were employed to explore differences in the demographic and clinical data (SPSS 20.0, Chicago, USA). Continuous variables were presented as mean ± SD. The threshold for statistical significance was defined as *P* < 0.05.

### Analysis of Covariance and Correlation Analyses

A two-way factorial analysis of covariance (ANCOVA: MDD, CACNA1C rs11832738 G allele) was performed using AlphaSim correction (FWHM = 4 mm, with 61 × 73 × 61 mm^3^ whole brain mask, which yielded a corrected threshold of *P* < 0.001, cluster sizes ≥6 mm^3^), by controlling for age, sex, education, and head motion. Subsequently, numerical representation of the mean ALFF strength of each significant cluster was displayed to reflect ALFF alterations among groups.

To further identify the clinical significance of the altered ALFF, the average ALFF strength of each significant region observed in the ANCOVA analysis was extracted. Partial correlation analysis was employed to detect the relationship between the average regional ALFF strength from the interactive effect and depressive severity or therapeutic effect (2-week/8-week) as well as the genotype of rs11832738 (GG, GA, and AA genotypes with scores of 1, 0.5, and 0, respectively) in patients with MDD after controlling for covariates including sex, age, education, and duration of illness. The threshold of statistical significance was *P* < 0.05.

### Mediation Analyses

We performed mediation analyses to further determine whether ALFF strength could mediate the association between CACNA1C rs11832738 and MDD. This approach was based on a standard three-variable mediation model and was in line with the current most widely accepted mediation analysis technique (19, 20). A detailed description of the method is provided in the **Supplementary Material**.

## Results

### Demographic and Clinical Data

Demographic and clinical data are shown in **Table 1**. There were no significant differences in age, sex, or education among the different allele groups in the MDD and NC groups. There were no significant differences in age of onset, duration of illness, number of episodes, family history, HAMD-24 score (0-week/2-week/8-week) or HAMD-24 reductive rate (2-week/8-week) among the different allele groups in the MDD group.

**Table 1:** Demographic and clinical data of different allele groups in the MDD and NC groups.

	MDD (n = 116)		NC (n = 66)* *		*P1*	*P2*	*P3*
	GG/GA (n = 61)	AA (n = 55)	GG/GA (n = 26)	AA (n = 40)			
Sex (M/F)	32/29	22/33	11/15	20/20	0.179	0.541	0.957
Age (years)	45.10 ± 14.10	43.82 ± 13.82	40.35 ± 13.52	42.18 ± 13.75	0.623	0.579	0.155
Education (years)	10.18 ± 4.65	8.89 ± 4.33	12.50 ± 4.48	11.38 ± 4.64	0.126	0.244	0.071
Age of onset (years)	40.70 ± 14.81	38.84 ± 15.02	—	—	0.605	—	—
Duration of illness (months)	53.17 ± 80.38	60.33 ± 87.17	—	—	0.881	—	—
Number of episodes	2.05 ± 1.54	2.34 ± 2.37	—	—	0.539	—	—
Family history (Y/N)	5/56	6/49	—	—	0.619	—	—
HAMD-24 (0 w)	31.20 ± 7.08	31.24 ± 4.77	1.19 ± 1.96	1.13 ± 1.96	0.972	0.822	0.000
HAMD-24 (2 w)	15.85 ± 7.80	15.82 ± 15.76	—	—	0.979	—	—
HAMD-24 (8 w)	4.72 ± 5.40	3.78 ± 4.51	—	—	0.350	—	—
Reductive rate (2 w)	0.51 ± 0.18	0.50 ± 0.17	—	—	0.762	—	—
Reductive rate (8 w)	0.84 ± 0.18	0.88 ± 0.15	—	—	0.242	—	—

MDD, major depressive disorder; NC, normal control; HAMD-24, Hamilton Depression Scale-24; M/F, male/female; Y/N, yes/no. P1, comparison between the GG/GA and AA genotypes in the MDD group; P2, comparison between the GG/GA and AA genotypes in the NC group; P3, comparison between the MDD and NC groups.

### ANCOVA Analysis

**Figure 1:**
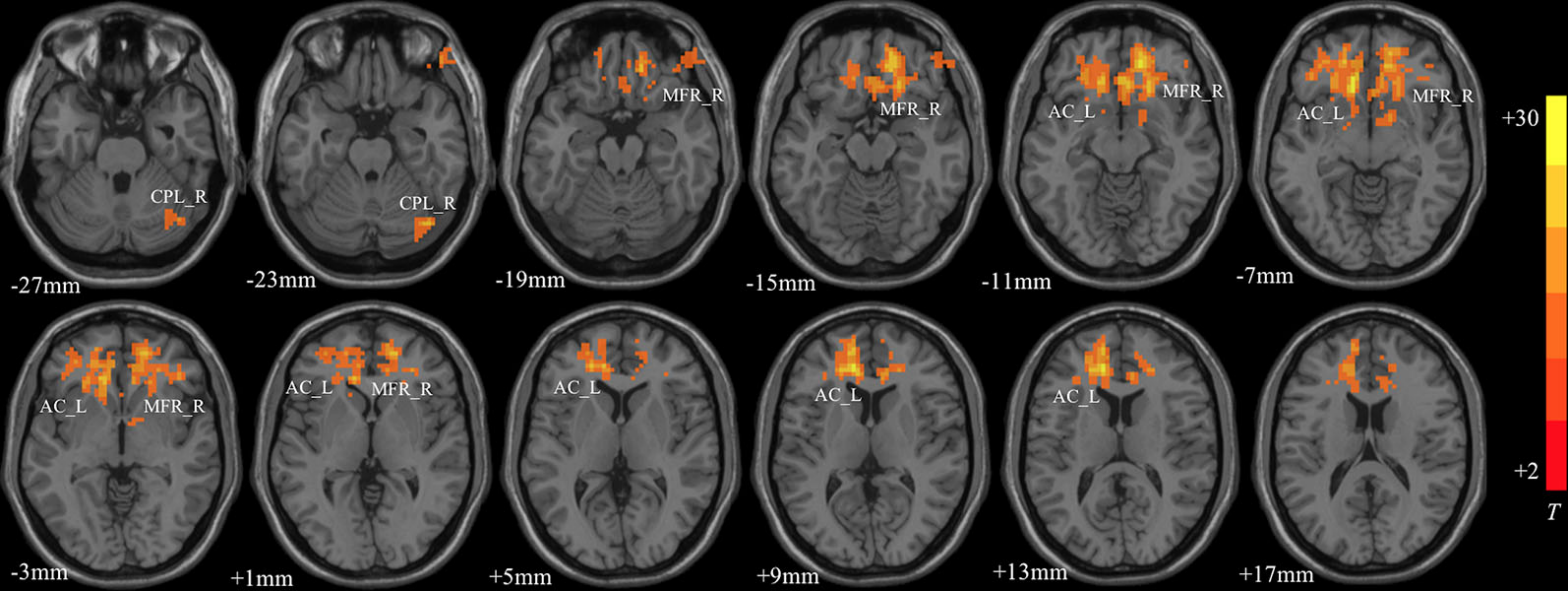
The main effect of MDD on regional ALFF distributed in the three brain areas: the right medial frontal gyrus (MFG_R), left anterior cingulate cortex (ACC_L), and right cerebellum posterior lobe (CPL_R; corrected *P* < 0.001, determined by AlphaSim with voxel number ≥6 for multiple corrections). The color bar indicates the display window for the threshold t-value maps. More intense colors indicate higher ALFF values.

**Figure 2:**
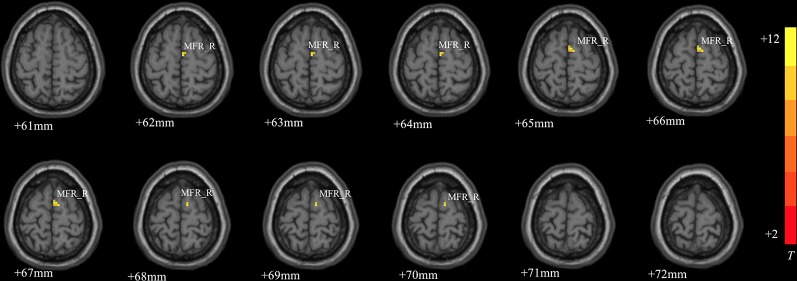
The interactive effect of gene and MDD on the ALFF of the right medial frontal gyrus (MFG_R; corrected *P* < 0.001, determined by AlphaSim with voxel number ≥6 for multiple corrections). The color bar indicates the display window for the threshold t-value maps. More intense colors indicate higher ALFF values.

MDD had a main effect on regional ALFF distribution in the three brain regions: the right medial frontal gyrus (MFG_R), the left anterior cingulate cortex (ACC_L), and the right cerebellum posterior lobe (CPL_R; **Table 2** and **Figure 1**). CACNA1C showed a significant interactive effect with MDD on the ALFF of MFG_R (**Table 3** and **Figure 2**). No main effect of the gene on regional ALFF was found (corrected *P* < 0.001, determined by AlphaSim with voxel number ≥6 for multiple corrections).

**Table 2:** Main effect of MDD on regional brain ALFF values.

Brain regions	BA	Voxel number	MNI coordinates			t-score
			X	Y	Z	
Right cerebellum posterior lobe	—	52	42	−72	−24	26.985
Anterior cingulate cortex	32	855	−12	27	−9	33.0669
Right medial frontal gyrus	10	736	15	45	−12	29.0273

The significance threshold was set at a corrected P < 0.001 (corrected with AlphaSim correction and cluster volume ≥6 voxels). ALFF, amplitude of low-frequency fluctuation; BA, Brodmann area; MNI, Montreal Neurological Institute.

**Table 3:** Interactive effect of MDD and gene on regional brain ALFF values.

Brain regions	BA	Voxel number	MNI coordinates			t-score
			X	Y	Z	
Right medial frontal gyrus	10	11	3	0	66	14.7147

The significance threshold was set at a corrected P < 0.001 (corrected with AlphaSim correction and cluster volume ≥6 voxels). ALFF, amplitude of low-frequency fluctuation; BA, Brodmann area; MNI, Montreal Neurological Institute.

### Correlation Analysis

**Figure 3:**
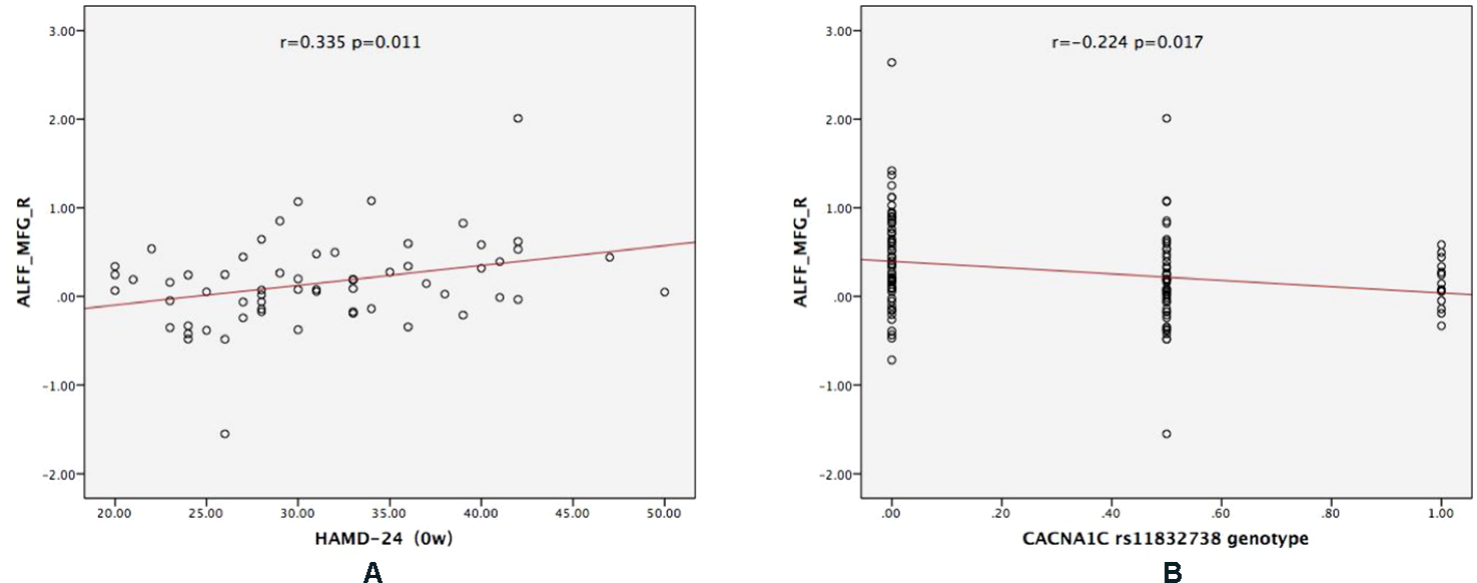
The results of the partial correlation analyses. **(A)** For CACNA1C G allele carriers, ALFF_MFG_R was significantly positively correlated with the baseline HAMD-24 score (*r* = 0.335, *P* = 0.011); **(B)** in the MDD group, CACNA1C rsll832738 was significantly negatively correlated with ALFF_MFG_R (*r* = −0.224, *P* = 0.017). ALFF_MFG_R: amplitude of low-frequency fluctuation of the right medial frontal gyrus.

For CACNA1C G allele carriers, the ALFF of MFG_R was significantly positively correlated with the baseline HAMD-24 score (*r* = 0.335, *P* = 0.011; **Figure 3A**). In the MDD group, CACNA1C rsll832738 was significantly negatively correlated with the ALFF of MFG_R (*r* = −0.224, *P* = 0.017: **Figure 3B**).

### Mediation Analysis

**Figure 4:**
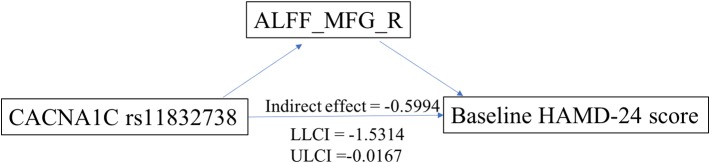
ALFF_MFG_R had a significant positive mediation effect between the CACNA1C rs11832738 polymorphism and the baseline HAMD-24 score. ALFF_MFG_R, amplitude of low-frequency fluctuation of the right medial frontal gyrus; LLCI, lower-limit confidence interval; ULCI, upper-limit confidence interval.

Exploratory mediation analysis revealed that the intrinsic ALFF in MFG_R had a significant mediation effect on the association between the CACNA1C rs11832738 polymorphism and baseline HAMD-24 score (**Figure 4**).

## Discussion

To the best of our knowledge, our study is the first to explore the relationship between the CACNA1C rs11832738 polymorphism and ALFF in patients with MDD. In the current study, we found that MFG_R was both the brain area that was mainly affected by the MDD as well as affected by rs11832738 and MDD interactively. Moreover, for CACNA1C G allele carriers, the ALFF of MFG_R was significantly positively correlated with depression severity. Finally, through further mediation analyses, we found that the relationship between the CACNA1C rs11832738 polymorphism and depression severity was mediated by the altered spontaneous activity of MFG_R.

The MFG, a region of the ventral lateral prefrontal cortex, is responsible for the top-down regulation of emotional processing and numerous cognitive functions, such as decision-making, working memory, and attentional processing, and its contribution to MDD has been documented (21, 22). Compared to the left side, MFG_R could modulate the cognitive shift between the internal and external environments due to the link between the ventral attention network and the dorsal attention network (21), and lesions in this area could lead to negatively biased attention in patients with MDD (23, 24). Importantly, considerable research has, to date, supported the idea that negatively biased attention predicts prolonged mood persistence in patients with MDD and future increases in depression severity (24–26). A recent study also suggested that abnormal spontaneous activity (reflected by ALFF) of MFG_R predicts improvement in depressive symptoms (27). Our study found a significant correlation between spontaneous activity of MFG_R and depression severity. Therefore, MFG_R might have a closer relationship with MDD than does the left MFG.

Previous studies have suggested that the CACNA1C SNP can alter the function of the frontal cortex (11) and that the knockdown of CACNA1C in the frontal cortex has an antidepressant-like effect (28), suggesting that CACNA1C might affect depression by altering frontal lobe function. Our study is consistent with these studies and showed that the effect of this gene on the severity of depression is mediated by changes in the spontaneous activity of MFG_R.

The underlying mechanism may be related to the contribution of Cav1.2 signaling. Cav 1.2 is encoded by CACNA1C and is the predominant calcium channel in the brain (accounting for approximately 90% of calcium channels), acting as the main pathway that mediates the entry of calcium into cells (29). Thus, it is of vital importance to intracellular signaling pathway activity, gene expression, synaptic plasticity, and neuronal and dendritic development, suggesting that perturbations in Cav1.2 signaling can lead to depressive phenotypes (28, 29). Cav1.2 modulates the calcium-dependent genes, including brain-derived neurotrophic factor and B-cell lymphoma 2, the protein products of which have been demonstrated to have neurotrophic and neuroprotective effects in the context of corticolimbic frontotemporal structures and functions (30). Although not specifically about rs11832738, previous studies have suggested that SNPs could influence altered CACNA1C gene expression, which in turn would lead to the dysfunction of Cav1.2 and altered neural activity (12). Our results suggest that rs11832738 might contribute to depression severity through altered CACNA1C gene expression; however, this requires further research.

In addition to MFG_R, we found that the right CPL and ACC were also affected by MDD. The ACC is responsible for regulating stress and processing emotion and has repeatedly been shown to play an important role in MDD (31–33). The cerebellum is traditionally thought to be involved only in motor function, but in recent years, it has been increasingly recognized that the cerebellum is important for cognition and emotion (34). Therefore, an increasing number of studies have suggested that the cerebellum participates in the pathophysiological mechanism of depression (35). Moreover, a meta-analysis indicated that posterior and lateral cerebellar regions regulate cognition and emotion, whereas the anterior lobe is mainly involved in motor function (36). This is consistent with our results, demonstrating that the posterior lobe is associated with MDD.

There were two main limitations to our study. First, as with many other rs-fMRI studies, there was no way to constrain the subjects' thoughts during scanning, especially for MDD patients, making the cross-sectional resting ALFF comparison more difficult as there may already be differences in the patients' ‘resting state'. Second, our data were collected from two sites, similar to many multicenter studies (37), and the site effect was an important problem which should not be ignored. However, to ensure the same scanning parameters and minimize the differences between the two sites, an indicated person (Chunming Xie) was invited to debug the MRI scan parameters of the two sites before we collected the data. One advantage of our study setup was the use of mediation analysis, which provided direct evidence of the neural basis of MDD.

## Conclusions

This study provides initial evidence for CACNA1C genotype-related alterations in brain function among patients with MDD and contributes to a better understanding of the neurobiological mechanisms underlying MDD. This adds to the current state of knowledge regarding the effects of CACNA1C on brain function. Our study, together with previous studies, suggests that calcium channel dysfunction may, in part, contribute to the genetic etiology of MDD through alterations in the functional activity of the brain. In other words, MDD may be, in part, an ion channelopathy.

However, the present findings require replication with larger samples. Moreover, an important step in determining whether specific effects of the CACNA1C genotype exist in subgroups of patients with MDD is the investigation of different patient samples.

## Data Availability Statement

The datasets analyzed in this article are not publicly available. Requests to access the datasets should be directed to 1360809788@qq.com.

## Ethics Statement

The studies involving human participants were reviewed and approved by the Medical Ethics Committee for Clinical Research of Zhongda Hospital Affiliated to Southeast University. The patients/participants provided their written informed consent to participate in this study.

## Author Contributions

XL was responsible for data collection, data processing and writing. YYi and ZH were responsible for data collection. CX, HaZ, HoZ and ZZ were responsible for data quality testing. YYu was responsible for the final examination of the paper.

## Funding

This work was supported by the National Key Research and Development Program of China (2016YFC1306702), the National Natural Science Foundation of China (81971277), and the Scientific Research Foundation of Graduate School of Southeast University (YBPY1890).

## Conflict of Interest

The authors declare that the research was conducted in the absence of any commercial or financial relationships that could be construed as a potential conflict of interest.
