# Supplementary material for: CACNA1C Gene rs11832738 Polymorphism Influences Depression Severity by Modulating Spontaneous Activity in the Right Middle Frontal Gyrus in Patients With Major Depressive Disorder
Source: Front Psychiatry. 2020 Feb 25;11:73. doi: 10.3389/fpsyt.2020.00073 (PMC7052844; doi:10.3389/fpsyt.2020.00073)
Supplement: Supplementary file 1 [file Presentation_1.pdf]

SUPPLEMENTARY MATERIAL

Title: CACNA1C Gene rs11832738 Polymorphism Influences Depression Severity by Modulating Spontaneous Activity in the Right Middle Frontal Gyrus in Patients With Major Depressive Disorder

Mediation analysis

The classic mediation model was selected and then Sobel test was used to confirm the significance of the mediator if the weighted coefficient (a or b) was not significance. Three steps regression models were constructed, as shown below:

Y = *c*X + *e*1 (1)

M = *a*X + *e*2 (2)

Y = *c'*X + *b*M + *e*3 or $z = \frac{ab}{\sqrt{(b^2 \mathrm{SE}_a^2) - (a^2 \mathrm{SE}_b^2)}}$ (3)

Where X is the independent variable (CACNA1C rs11832718 polymorphism), Y is the dependent variable (depression severity or therapeutic effect), M is the mediator (ALFF strength), a is the regression coefficient for the relationship between CACNA1C rs11832718 polymorphism and ALFF strength in distinct region, b is the regression coefficient for the relationship between ALFF strength in distinct region and depression severity or therapeutic effect, c is the regression coefficient for the relationship between CACNA1C rs11832718 polymorphism on depression severity or therapeutic effect. z and c' represent the effect of CACNA1C rs11832718 polymorphism on depression severity or

therapeutic effect while controlling for the indirect effect. SEa is the standard error of the relationship between CACNA1C rs11832718 polymorphism and the ALFF strength and SEb is the standard error of the relationship between ALFF strength and depression severity or therapeutic effect.

In this analysis, four conditions for establishing mediation are: 1) c must be significant; 2) a and b are significant; 3) c’ < c (in absolute value, partial mediation) or c’ is insignificant (full mediation); 4) if a or b is insignificant, the Sobel test Z must be significant. If the mediator is existed, we using ratio indirect to present the strength of mediation ((a*b)/c).
